# A Novel Method to Repair Thin Endometrium and Restore Fertility Based on Menstruation-Derived Stem Cell

**DOI:** 10.1007/s43032-024-01458-2

**Published:** 2024-01-31

**Authors:** Kai Chen, Huiru Wang, Xin Zhao, Jingxin Wang, Qi Jin, Xianhong Tong, Shengxia Zheng

**Affiliations:** 1 Reproductive Medicine Center & Department of Obstetrics and Gynecology, The First Affiliated Hospital of USTC, Division of Life Sciences and Medicine, University of Science and Technology of China, Hefei, 230001 Anhui China; 2 Wannan Medical College, Wuhu, 241002 Anhui China

**Keywords:** Thin endometrium, Menstrual blood stem cells (MenSCs), Decidualization, Stem cell therapy, Animal model

## Abstract

Thin endometrium (TE), which mainly occurs as a result of severe damage to the endometrial basalis, is one of the prominent etiologies of menstrual abnormalities, infertility, and recurrent miscarriage in women. Previous studies have demonstrated that mesenchymal stem cells (MSCs) are considered ideal cells with multipotency for regenerative medicine and exhibit therapeutic effects on TE through their cellular secretions. However, there is limited research on strategies to enhance MSC secretion to improve their therapeutic efficacy. Herein, we isolated menstrual blood–derived mesenchymal stem cells (MenSCs) from menstruation and transformed them into decidualized stromal cells (DSCs), which are specialized cells with enhanced secretory functions. To assess the therapeutic potential of DSCs compared to MenSCs, we conducted a series of experiments in cells and animals. The results demonstrated that DSCs exhibited changes in morphology compared to MenSCs, with a decrease in cell proliferation but a significant improvement in secretion function. Furthermore, DSCs facilitated the restoration of endometrial thickness and increased the number of glands and blood vessel formation. Most importantly, the pregnancy rates in rats were effectively restored, bringing them closer to normal levels. These findings greatly contribute to our understanding of stem cell therapy for TE and strongly suggest that DSCs could hold significant promise as a potential treatment option for TE.

**Supplementary Information:**

The online version contains supplementary material available at 10.1007/s43032-024-01458-2.

## Introduction

Human endometrium can be divided into approximately two-thirds of the functional layer located in the upper region and one-third of the basal layer situated in the lower basal layer [1]. The periodic shedding of the functional layer, regulated by fluctuating levels of estrogen and progesterone, facilitates endometrial self-renewal [2]. Normal endometrium plays a crucial role in the establishment and maintenance of pregnancy [3]. However, invasive procedures performed within the uterine cavity, such as curettage after abortion, can result in damage to the basal layer and subsequently lead to thinning of the endometrium (TE) [4].

TE can significantly impair endometrial receptivity, leading to embryo implantation failure or post-pregnancy miscarriage [5]. Therefore, restoring TE to its normal thickness is essential for the successful establishment and maintenance of pregnancy [6, 7]. Currently, the primary clinical approach for treating TE involves stimulating endometrial growth through estrogen administration following adhesions under hysteroscopy [8]. However, traditional hysteroscopic treatment cannot fully achieve the desired effect and is often accompanied by a high recurrence rate. To prevent re-adhesion after hysteroscopy separation of adhesion, researchers have explored the use of intrauterine devices (IUDs), balloons, and sodium hyaluronate to prevent adhesion after surgery, but still could not fundamentally solve the problem [9, 10]. Thus, treatment of TE remains a thorny problem, and future investigations are imperative to enhance endometrium thickness and pregnancy outcomes in patients with TE.

Mesenchymal stem cell (MSC) has attracted more and more attention in the treatment of TE, for MSC shows great potential in promoting cell proliferation, immune regulation, and angiogenesis in tissue repair [11]. It has been proved that the main cause of TE is the reduction in the number of basal layer stem cells of the endometrium [12]. Researchers extracted MSC from the endometrium of both TE patients and normal women, respectively, for comparative analysis. The results showed that the MSC derived from TE endometrium had lower angiogenesis and cell proliferation capacity [13]. Consequently, restoring the population of basal stem cells plays an important role in improving endometrium thickness and enhancing pregnancy rates in TE patients.

Endometrial decidualization refers to the transformation of endometrial stromal cells into specialized secretory decidualized cells with hormonal changes, which plays an indispensable role in successful embryo implantation and early pregnancy maintenance [14]. The development of endometrial decidualization is the foundation of pregnancy as it promotes angiogenesis and immunomodulation [15]. It has been shown in recent studies that abnormal endometrial decidualization is strongly associated with early biochemical pregnancy and abortion, and improvement of endometrial decidualization will play a crucial role in embryo implantation and successful pregnancy [16]. However, as decidualization occurs in the female endometrium, few researchers have recapitulated this process in vitro using menstrual blood-derived mesenchymal stem cells (MenSCs). It remains unclear whether decidualized stromal cells (DSCs) play a definite role in repairing TE.

In this study, we first induced MenSCs into DSCs in vitro and subsequently compared the morphological and functional characteristics between these two cell types. Secondly, the two types of cells were compared in terms of their effects on enhancing endometrial thickness, number of glands, and angiogenesis. Finally, the effects on fertility recovery of TE rats were observed by counting the number of embryos. Altogether, this study provides a new idea for the clinical treatment of infertility patients caused by TE.

## Materials and Methods

### Animals

Eight- to 10-week-old Sprague-Dawley female rats weighing 160–200 g were used in all experiments, and all rats were purchased from SLAC Laboratory Animals (Shanghai, China). The animals had free access to water and food and were maintained in a feeding room on a 12-h light and 12-h dark regimen with an average temperature of 22 °C and 70 to 80% relative humidity. All the procedures were approved by the Institutional Animal Care and Use Committee at the First Affiliated Hospital of USTC (code no. 2022-N(A)-119).

### Isolation of Menstrual Blood Stem Cells (MenSCs)

Menstrual blood donors aged 25–40 years old, who were diagnosed without any reproductive system-related diseases (*n*=3). All donors gave consent, and all procedures were approved by the Ethics Committee of First Affiliated Hospital of USTC (IRB code NO. 2021KY015). MenSCs were isolated using human lymphocyte isolation solution (abs930, Absin, China). The cells were routinely cultured in Dulbecco’s Modified Eagle’s Medium (DMEM,1-051-1ACS, BI, Israel) supplemented with 100 U/mL penicillin, 100 μg/mL streptomycin (BL505A, Biosharp, China), and 10% fetal bovine serum (FBS, F7524, Sigma, Germany) at 37 °C under a humidified atmosphere containing 5% CO2. The culture medium was changed every 3–4 days until adherent cells reached a confluence of approximately 90%; then, the cells were passaged using 0.05% trypsin-EDTA solution (25300-062, Gibco, USA).

### Immunofluorescence

For paraffin tissue sections, deparaffinization was performed using a dewaxing solution (G1128, Servicebio, China) followed by rehydration in decreasing concentrations of ethanol. For cells on culture dishes, the supernatant was discarded and the cells were fixed with paraformaldehyde (G1101, Servicebio, China) at room temperature for 20 min. After antigen retrieval and blocking of endogenous peroxidases, the samples were incubated in a wet box overnight at 4 °C with antibodies raised against Vimentin (VIM, SC-6260, Santa Cruz, USA, 1:200), cytokeratin7 (CK 7, 15539-1-AP, Proteintech, China, 1:200), CD31 (28083-1-AP, Proteintech, China, 1:200), and OCT-4 (ab19857, Abcam, USA). After overnight incubation, the samples were washed with PBS and incubated with Alexa Fluor 488 or Alexa Fluor 594 secondary antibody (Jackson labs, USA, 1:200) for 3 h at room temperature. This was followed by another wash in PBS and nuclear staining conducted with DAPI (1155MG010, BioFroxx, Germany, 1:1000). All procedures were in dark conditions. Images were collected by ECHO Revolve FL (ECHO, USA). All immunofluorescence tests were repeated at least three times.

### Identification of MenSCs

Flow cytometry was conducted using the Human MSC Analysis Kit (562245, BD Biosciences, USA). Briefly, MenSCs were detached from the culture dish using Accutase (A6964, SIGMA, USA) at passage 3, and then resuspension in FACS buffer at a concentration of 2 × 10^4^ cells/20 μL. Then, they were incubated with the respective antibodies (1:200) in the dark at room temperature for 20 min: IgG1-PE, IgG1-FITC, IgG1-PC5.5, CD44-PE, CD73-APC, CD90-FITC, CD105-PC5.5, and CD11B/34/79a-PE. Then, the cells were washed with FACS buffer and centrifuged at 600 rpm for 5 min. The cells were resuspended with 400 μL of FACS buffer. The antibody-labeled cells were analyzed with a BD FACSAriaII cell sorter (*n*=3).

We used a Human MSC Functional Identification kit (SC006, R&D systems, USA) to evaluate the ability of MenSCs to differentiate into multiple mesenchymal lineages (adipogenic, osteogenic, and chondrogenic). Briefly, the MenSCs were cultured in a 24-well plate and incubated with differentiation medium to induce the differentiation process. Following differentiation, the cells were fixed with paraformaldehyde. Ultimately, differentiation outcomes (*n*=3) were assessed through immunofluorescence staining of osteocalcin as an osteocyte marker, FABP-4 as an adipocyte marker, and aggrecan as a chondrocyte marker (undifferentiated MenSCs as negative control).

### Decidualization of MenSCs

Upon reaching 70% confluence in the petri dish, the MenSCs were subjected to a medium change with differentiation media. Specifically, the differentiation medium was supplemented with 10 nM/L 17-β-E2 (abs47006987, Absin, China), 1 uM/L MPA (abs44122880, Absin, China), and 0.2mM/L cAMP (D0627, Sigma, USA). After 14 days, the induction of decidualization was brought to a halt (*n*=3).

### Cell Proliferation Curve

The cells were seeded at a density of 100,000 cells per well in 6-well plates (*n*=3). Adherent cells were then digested and counted every 2 days to generate the cell proliferation curves based on the cell count.

### VEGF-A Quantification

Media conditioned by MenSCs and DSCs were collected after 24 hours (*n*=3). The concentration of VEGF-A (RK00023, ABclonal, China) and PRL (KE00172, Proteintech, China) was quantified using a commercially available enzyme-linked immunosorbent assay kit. Firstly, samples were added and incubated at 37 °C for 2 h. Next, antibodies were added and incubated at 37 °C for 1 h. Streptavidin-horseradish peroxidase was then added for another incubation at 37 °C for 40 min. Finally, after adding the chromogenic solution and the terminating solution, the absorbance was measured, and a standard curve was constructed to determine the concentration of the sample under investigation.

### Endothelial Cell Scratch Healing Assay

A total of 2 × 10^5^ human umbilical vein endothelial cells (HUVECs, Oricell) at passage 4 were seeded in a 6-well plate. Once the cells reached 100% confluence, scratches were made by a 200-μL sterile pipette tip perpendicular to the marking line. The culture medium was changed into conditioned medium (MenSCs and DSCs for the experimental group and DMEM for the control group). All wells were imaged at 0 h and 24 h after creating a scratch. ImageJ software was used to calculate the average area between cells (*n* = 3).

### Determination of Estrus

A sterile cotton swab was immersed in normal saline to collect the rat vaginal swab (*n*=9). The swab should be gently rotated and rolled against the vaginal wall before being removed. The collected cells are then delicately transferred onto a dry glass slide by gently sliding the swab across the surface, followed by microscopic examination for identification of cell composition. When the vaginal swab contained more anucleated keratinized epithelial cells and fewer neutrophils, it indicated that the rats’ uterus lining was thicker during this period.

### Establishment of the Rat Thin Endometrium (TE) Model

Rats in estrus were selected based on vaginal secretions and then anesthetized with Zoletil 50 (Virbac, France) via intramuscular injection at a concentration of 0.1 mL/100 g. Following shaving, the lower abdomen was disinfected with iodophor. A midline incision was made across the skin and muscles to expose the uterus. A precise longitudinal incision measuring 5 mm in length was made near the uterine wall, close to the vaginal opening. Upon entering the uterine cavity, gentle rubbing was performed with the eye forceps for a total of ten repetitions, resulting in a collective total of 40 repetitions. Subsequently, the uterine cavity was washed with 5 mL of normal saline thoroughly. Then, the uterus was sutured using 6-0 absorbable sutures, followed by suturing of the muscles using 5-0 absorbable sutures. Finally, the skin was sutured using non-absorbable sutures. All rats were randomly assigned to the group.

### Histological Analysis

Hematoxylin and eosin (H&E) and Masson staining were employed in the evaluation of rat endometrial tissue (*n*=6). Paraformaldehyde was fixed at room temperature for at least 24 h and then embedded in paraffin after removing the rat uterus. The sections were then cut into a thickness of 10 μm. The endometrial thickness was measured from the luminal epithelium to the smooth muscle layer with imaging. The two perpendicular lines are averaged, and the average of the three measured slices was taken.

### Quantitative Real-Time Polymerase Chain Reaction

Total RNA was extracted from the adherent cells or excised uterine tissues by using the RNA-Quick purification kit (RN001, Esscience, China), and the reverse transcription was employed using RT reagent kit (R-323-01, Vazyme, China). Cham Q Universal SYBR qPCR Master Mix (Q511-02, Vazyme, China) was used for quantitative RT-PCR reaction. The primers used in this study are listed in Table 1. Quantitative RT-PCR was performed by LightCycler 96 Instrument (Roche, USA), with the following program set to 95 °C 30 s, 95 °C 10 s, 60 °C 30 s, 40 cycles, 95 °C 15 s, 60 °C 60 s, 95 °C 15 s. GAPDH was used to normalize the relative levels of the gene (*n*=3).

**Table 1:** Primes used in this study

Prime	Forward prime	Reverse prime
Human-derived primes		
GAPDH	ACACCATGGGGAAGGTGAAG	GTGACCAGGCGCCCAATA
PRL	CAAAGGATCGCCATGGAA	CACAGGAGCAGGTTTGACAC
IGFBP-1	TTTTACCTGCCAAACTGCAACA	CCCATTCCAAGGGTAGACGC
LIF	CCAACGTGACGGACTTCCC	TACACGACTATGCGGTACAGC
HOXA10	CTCGCCCATAGACCTGTGG	GTTCTGCGCGAAAGAGCAC
OCT-4	GCTGGAGCAAAACCCGGAGG	TCGGCCTGTGTATATCCCAGGGTG
Rat-derived primes		
GAPDH	TTCCTACCCCCAATGTATCCG	CATGAGGTCCACCACCCTGTT
VEGF	ACATCTTCAAGCCGTCCTGTGTGC	AAATGGCGAATCCAGTCCCACGAG

### Fertility Test

Eight- to 10-week-old Sprague-Dawley female rats were used in the therapy experiment. Group 1 (6 VS 6) was the sham operation group on the left and the control (without any treatment) on the right. In group 2, the left uterus was the sham-operated side, and the right side was the TE side (15 VS 15). In group 3, the left uterus was the sham-operated side, and the right side was the TE+MenSCs therapy side (15 VS 15). In group 4, the left uterus was the sham-operated side, and the right side was the TE+ DSCs therapy side (15 VS 15). In all cell-based therapy experiments, a total of 1 × 10^6^ cells were suspended in 50 μL of DMEM. In the control group, 50 μL of DMEM was injected into the uterine cavity. On the 14^th^ day after treatment, the female rats were housed together with healthy male rats at a ratio of 2:1. The discovery of the vaginal plug-in female rat is considered as day 0 of pregnancy [17]. The pregnant rats were sacrificed on gestation days 14–18, and the number of embryos on both sides was counted.

### Statistics

Statistical analysis was performed using GraphPad Prism 7.0 (San Diego). The results are shown as mean ± SEM. In this experiment, the one-way ANOVA test was used in the statistical analysis of multiple comparisons between groups. The two-tailed paired *t*-test was used in the comparative statistical analysis of the two groups. *p < 0.05, **p < 0.01, ***p < 0.001 are considered statistically significant.

## Results

### Isolation and Identification of MenSCs

**Fig. 1:**
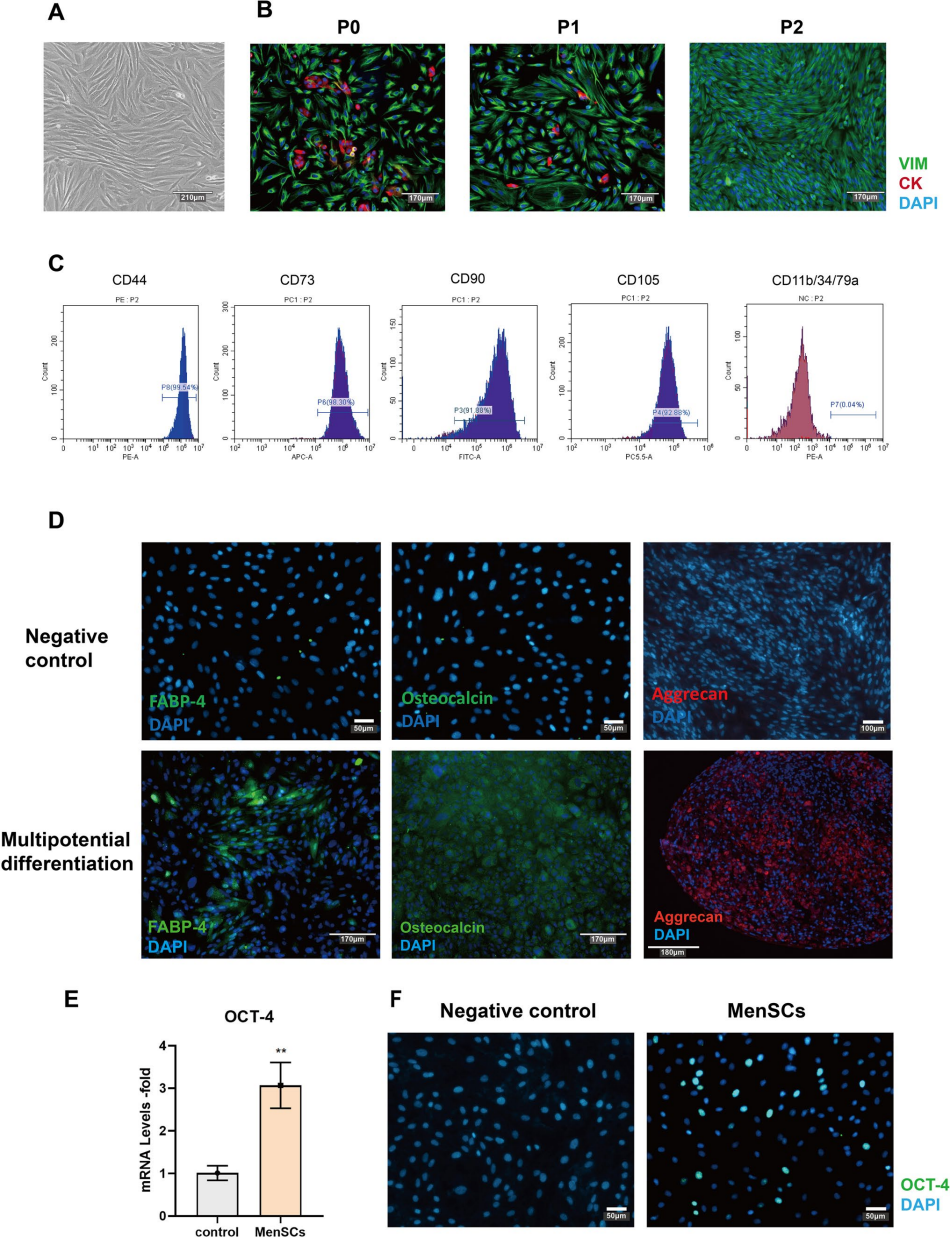
Isolation and identification of MenSCs. **A** The morphology of MenSCs in the bright-field. Scale bar: 210 μm. **B** Cell composition in primary cell (P0), passage 1 (P1), and passage 2 (P2). Green represents the stromal cell marker (VIM), red represents the epithelial cell marker (CK7), and blue represents the nuclear marker (DAPI). Scale bar: 170 μm. **C** Isolated MenSCs at passage 4 were used for flow cytometry analyses, and the values represent the percentage of positive cells among all cells. CD44 (99.54%), CD73 (98.30%), CD90 (91.88%), CD105 (92.88%), CD11b/CD34/CD79a (0.04%). (**D**) Immunofluorescence staining verified the multipotent differentiation ability of the MenSCs, representing the ability of adipogenesis (FABP-4), osteogenesis (osteocalcin), and chondrogenic (aggrecan) differentiation, negative control was undifferentiated MenSCs. Negative control scale bar: 50 μm. Multipotential differentiation scale bar: 170 μm. **E** OCT-4 gene expression in the MenSCs, the control is fibroblast cells. **F** OCT-4 protein in the fibroblast (negative control) and MenSCs. Scale bar: 50 μm

Menstrual blood–derived mesenchymal stem cells (MenSCs) were isolated from female menstrual blood and cultured in the petri dish, exhibiting a fibroblast-like spindle morphology (Fig. 1A). The endometrium typically consists of epithelial cells, stromal cells, vascular endothelial cells, and immune cells [18]. Immunofluorescence was then performed to analyze the cell composition between different passages. There were stromal and epithelial cells in P0 and P1, but epithelial cells disappeared at P2 with the application of DMEM (Fig. 1B). Cells after P2 were used for all the subsequent experiments to minimize any potential confounding effects from other cell types.

To evaluate the mesenchymal stem cell (MSC) properties of the MenSCs, we performed flow cytometry analysis to examine the cell surface marker of MenSCs at P3 [19]. More than 90% of cells were positive for CD44, CD73, CD90, and CD105, and less than 1% of MenSCs were positive for other cell surface markers, such as CD11b, CD34, and CD79a (Fig. 1C). In vitro differentiation experiments also proved that the MenSCs can be induced into osteoblasts, adipocytes, and chondrocytes, respectively (Fig. 1D), demonstrating their multipotent differentiation.

**Fig. 2:**
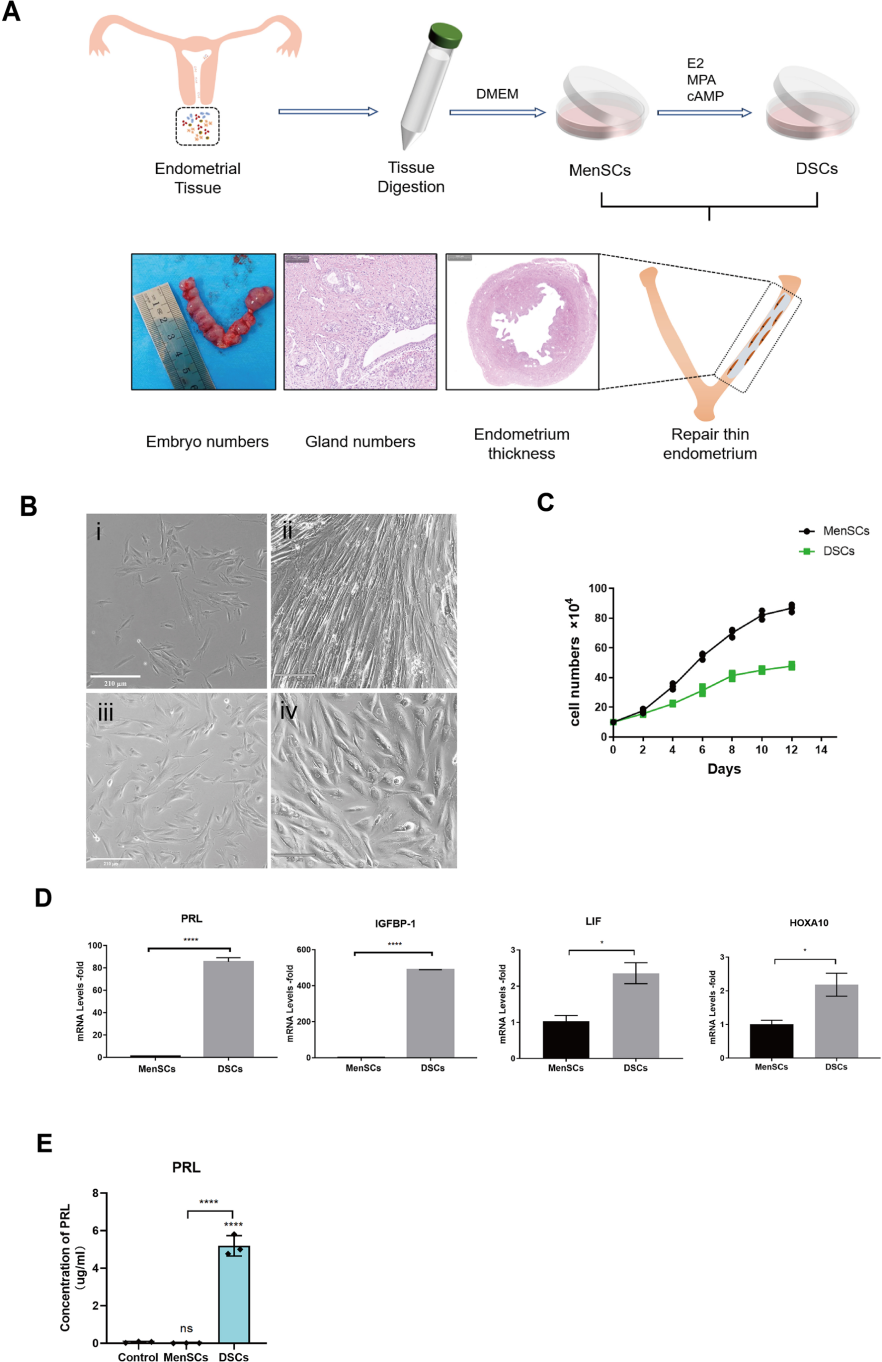
MenSCs induced into decidual stromal cells. **A** Schematic overview of MenSCs/DSCs treatment for thin endometrium. **B** The morphology of MenSCs and decidual stromal cells (DSCs) in the bright-field: (i) Cell morphology on the 1st of the control group, (ii) cell morphology on the 14th of the control group, (iii) cell morphology on the 1st of the decidualization group, (iv) cell morphology on the 14th of the decidualization group, Scale bar: 210 μm. **C** Cell proliferation curve between MenSCs and DSCs. **D** Expression of decidual-related genes (PRL and IGFBP-1) and endometrial receptivity-related genes (LIF and HOXA-10) after MenSCs-induced decidualization. **E** The PRL concentration in the supernatant was measured in different groups, with the control group being the DMEM medium

Previous studies have shown that endometrial stem cells highly express the OCT-4 gene [20]. To investigate the expression of the OCT-4 gene in MenSCs, we compared it with fibroblasts. According to the results, it is evident that MenSCs express the OCT-4 gene at high levels and also exhibit substantial expression of the OCT-4 protein (Fig. 1E, F). These findings provide evidence for the origin of MenSCs from the endometrium (compared with fibroblast). The experimental procedure is summarized in Fig. 2A.

### Decidualization of MenSCs In Vitro

The P3 MenSCs were used to induce decidualization in vitro, resulting in a transformation of cell morphology from spindle-shaped to oblate (Fig. 2B). Apart from the morphological changes, the proliferative ability of decidualized stromal cells (DSCs) was found to be weaker compared to that of MenSCs (Fig. 2C). The expression levels of decidual-related genes (PRL and IGFBP-1) significantly increased in DSCs, along with an increase in endometrial receptivity-related genes (LIF and HOXA-10) (Fig. 2D). Furthermore, the ELISA experiment revealed higher levels of PRL protein in the DSC cell supernatant, indicating that DSC cells secrete a greater amount of PRL protein (Fig. 2E). These findings suggest significant functional differences between DSCs and MenSCs, as indicated by variations in cell morphology, proliferation capacity, and gene expression patterns.

### MenSCs and DSCs Secret More VEGF-A In Vitro

**Fig. 3:**
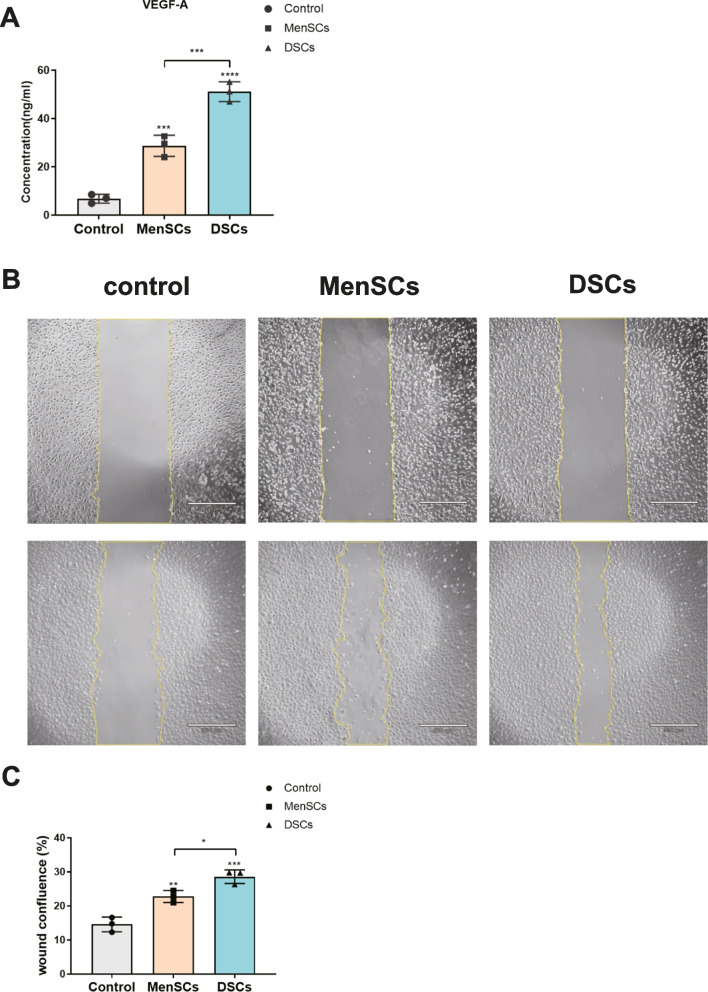
MenSCs and DSCs secret more VEGF-A in vitro. **A** The concentration of soluble VEGF-A in control (un-conditioned media), MenSCs (MenSCs supernatant), and DSCs (DSCs supernatant). **B** The impact of supernatants from different groups on the proliferation and migration of vascular endothelial cells was investigated. Scale bar: 530 μm. **C** Quantification of the wound healing assay results. Values are expressed as average ± SEM of three replicates

Researchers have proven that MSCs possess the ability to induce angiogenesis, primarily through the secretion of VEGF-A [21]. Therefore, we verified the secretion of VEGF-A by detecting the concentration of supernatant. Our results indicate that MenSCs and DSCs secreted more VEGF-A compared to the un-conditioned media. In addition, DSCs secrete twice as much VEGF as MenSCs (Fig. 3A). Subsequently, we found that the supernatant from the MenSCs and DSCs significantly promoted scratch closure of endothelial cells compared to the control (Fig. 3B, C). These results demonstrate that both DSCs and MenSC secrete VEGF and promote the proliferation and migration of vascular endothelial cells.

### The Therapeutic Effects of MenSCs and DSCs on TE Rats

**Fig. 4:**
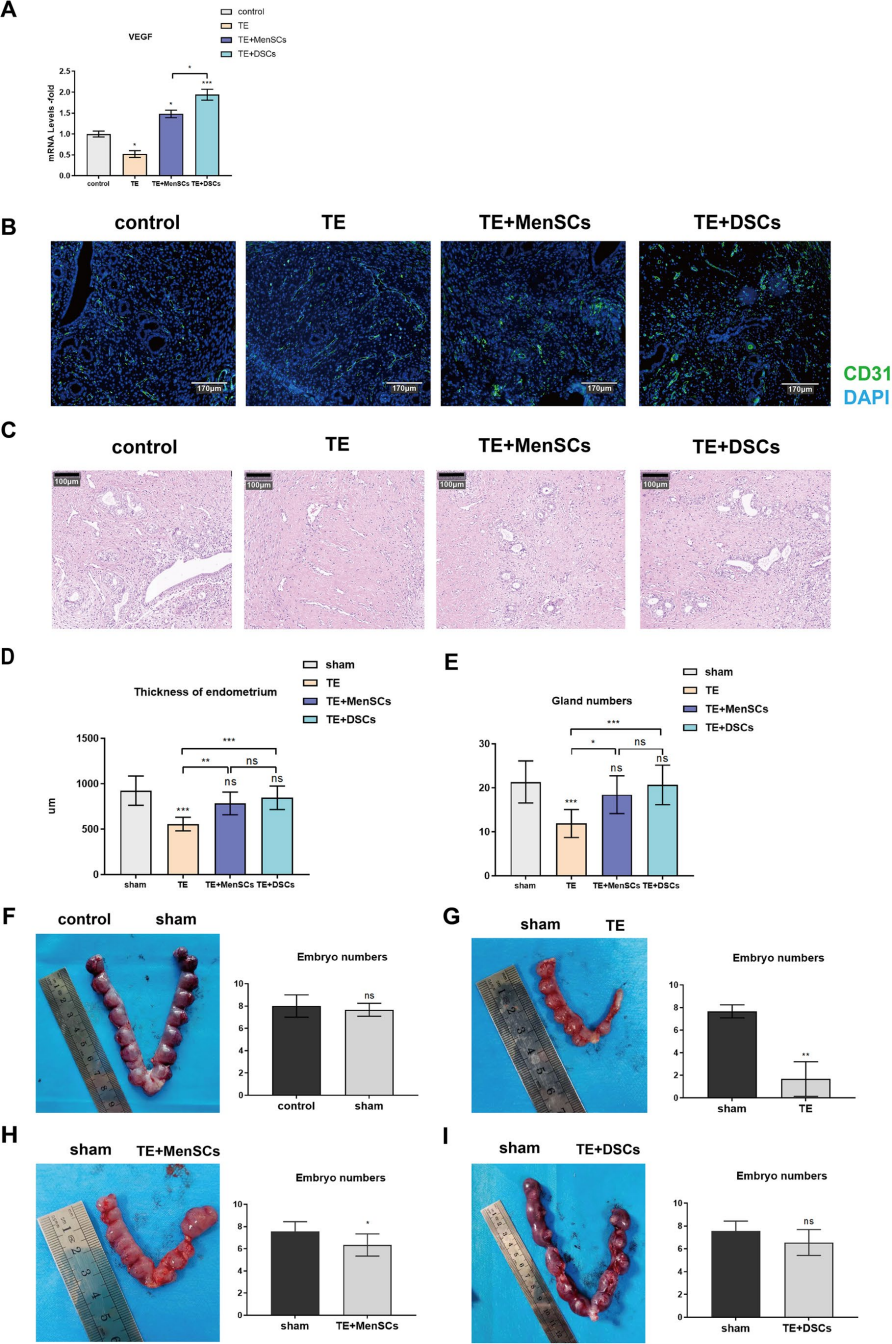
The therapeutic effects of MenSCs and DSCs on TE rats. **A** Expression of genes after treatment in different groups. VEGF-A: angiogenesis-related gene. **B** Expression of uterine vascular endothelial (CD31) in rats among different groups. Control, without any treatment; TE, rat model of thin endometrium; TE+MenSCs, MenSCs treat TE; TE+DSCs, DSCs treat TE. Scale bar: 170 μm. **C** H&E staining of rat uterine after different treatments. Scale bar: 100 μm. **D** Statistical analysis of endometrial thickness after different treatments. Values are expressed as average ± SEM of three replicates. **E** Statistical analysis of glands of the endometrium after different treatments. Values are expressed as average ± SEM of three replicates. **F** Image of embryo numbers on each side of the uterus after treatment (control vs sham), and statistical analysis of the embryo numbers. **G** Image of embryo numbers on each side of the uterus after treatment (sham vs TE), and statistical analysis of the embryo numbers. **H** Image of embryo numbers on each side of the uterus after treatment (sham vs TE+MenSCs), and statistical analysis of the embryo numbers. **I** Image of embryo numbers on each side of the uterus after treatment (sham vs TE+DSCs), and statistical analysis of the embryo numbers. *p < 0.05, **p < 0.01, ***p < 0.001

Before conducting the animal experiment, we determine whether the rat is in estrus based on its vaginal secretion [22]. When the vaginal secretion contained more anucleated keratinized epithelial cells, the uterus of the rats was thicker at this time, which was conducive to the establishment of the TE model (Supplement Fig. 1A, B). Compared to normal endometrium, thin endometrium exhibits significantly reduced gland thickness and number, as well as an increased fibrosis area (Supplement Fig. 1C-E). Additionally, the number of embryos successfully implanted is notably reduced (Fig. 4G). Taking all of this into consideration, we can conclude that our TE rat model has been successfully established.

Afterwards, we transplanted MenSCs and DSCs into the uterus of the rat for a duration of up to 14 days. Following this, we extracted the gene from the rat endometrium and discovered that both groups showed a significant increase in the VEGF-A gene expression, with DSCs demonstrating superior efficacy compared to MenSCs (Fig. 4A). Additionally, by IF staining, we observed a more pronounced angiogenic response following transplantation of MenSCs and DSCs, and treatment with DSCs has more new blood vessels than treatment with MenSCs (Fig. 4B). To evaluate the therapeutic efficacy of MenSCs and DSCs for TE, we examined the endometrial regeneration and fertility restoration after transplantation of MenSCs and DSCs. It was observed that the thickness of the endometrium and gland number recovered to a level comparable to the control group after 14 days of transplantation (Fig. 4E). The findings suggest that both MenSCs and DSCs exhibit beneficial effects on endometrium regeneration in the TE model, with no significant difference observed between the two groups.

Furthermore, we performed a fertility test to evaluate the therapeutic effects of MenSC and DSC transplantation on fertility restoration in the rat model of TE. Two weeks after the fertility test, we examined well-developed embryos in different treatment groups. There was no significant difference in the number of embryos between the sham and the control groups, indicating that the sham operation did not affect the pregnancy of the rats (Fig. 4F). The TE groups exhibit less than 20% of implanted embryos (Fig. 4G). Transplantation of MenSCs has shown a notable therapeutic effect on TE and significantly improves the pregnancy rate in rats (Fig. 4H). Nevertheless, there is still a significant statistical difference when compared to the sham-operated group on the opposite side. However, the transplantation of DSCs for treating TE proves to have a more pronounced therapeutic effect, resulting in a greater similarity in the number of embryos when compared to the contralateral sham operation group (Fig. 4I). These results suggest that both MenSC and DSC transplantation can promote endometrial regeneration and improve fertility. Most importantly, transplantation of DSCs has a better effect on restoring rat fertility than MenSCs.

Taken together, this study demonstrates that DSCs’ therapeutic effects were superior to MenSCs, probably by promoting angiogenesis, rather than due to their promotion of cell proliferation.

## Discussion

In this study, we have demonstrated a novel approach to improve the thickness and gland numbers of thin endometrium by inducing MenSC decidualization. Both MenSCs and DSCs were found to promote angiogenesis, but DSCs exhibited superior efficacy in promoting angiogenesis compared with MenSCs, thereby improving embryo implantation rates.

Infertility is a reproductive health problem all over the world, and the incidence of infertility patients has been increasing in recent years [23]. The normal endometrium plays an irreplaceable role in female conception. However, TE can severely damage the endometrial morphology and function, resulting in implantation failure [24]. Cell transplantation therapy has considered the most convincing treatment for moderate and severe TE [25]. Various tissue-derived stem cells have been used to treat TE, such as bone marrow, adipose, and umbilical cord [26–28]. However, there are some risks and ethical constraints associated with the acquisition of these MSCs (bone marrow and adipose). Menstruation-derived mesenchymal stem cells (MenSCs), which were first extracted from menstrual blood in 2007 [29], can be easily obtained through non-invasive surgery, thereby reducing many ethical controversies [30]. Most importantly, MenSCs possess the properties of MSC and have demonstrated powerful cell therapeutic capabilities in various diseases [24].

Based on the previous protocol, we first isolated the MenSCs from menstrual blood [12]. Before using flow cytometry to identify the characteristics of MenSCs, we employed immunofluorescence (IF) to determine the cell composition, for endometrium not only contains stromal cells but also a large number of epithelial cells, vascular endothelial cells, and immune cells [18]. According to our results, a small number of epithelial cells were observed within the stromal cell population in the first two passages. However, the epithelial cells disappeared by passage 3, possibly due to the application of a mesenchymal cell medium. Therefore, in the follow-up experiments, we all used the cells after the third generation to reduce the influence of other cells on the experimental results. Subsequent experiments demonstrated that these cells possess the characteristic traits of MSC and originate from the endometrium.

Decidualization refers to the morphological and functional changes of endometrial stromal cells in response to periodic fluctuations in hormone levels [31]. At present, there are primarily two methods for obtaining decidual cells: from decidual tissue derived from spontaneous abortion in vivo or by inducing endometrial-derived cell decidualization in vitro [32]. In this study, we induced MenSCs to undergo decidualized stromal cells (DSCs) in vitro, and the morphology of the DSCs changed obviously after decidualization, but the proliferation ability of DSCs decreased. Furthermore, the expression levels of decidual-related genes (PRL and IGFBP-1) and endometrial receptivity-related genes (LIF and HOXA10) all increased. We also detected a significant level of PRL protein secretion in the supernatant of decidualized cells through ELISA experiments. These findings indicate that MenSCs can be effectively differentiated into decidual cells in vitro, with distinct morphological and functional characteristics.

Endometrial decidual cells can promote angiogenesis and immunomodulatory effects, as well as positive regulatory effects on subsequent embryo implantation and placental development [14]. As we expected, the VEGF-A concentration in the supernatant of different groups increased, with DSCs secreting more VEGF-A compared to control and MenSCs. Animal experiments have fully verified its function in significantly promoting the formation of new blood vessels, as genes related to angiogenesis were found to be elevated 14 days after cell repair.

To directly observe the roles of MenSCs and DSCs in endometrial regeneration, the endometrial thickness and number of glands were measured after MenSC and DSC therapy. Similarly, the results indicated that both MenSCs and DSCs effectively promoted the thickness of the endometrium and regeneration of glands, with no significant difference observed between the two cell types. The change in endometrial receptivity is mainly reflected by the pregnancy rate [33]. However, based on the number of embryos, the therapeutic effect of DSCs was obviously better than that of MenSCs. Altogether, we concluded that DSCs could improve endometrial receptivity primarily by promoting angiogenesis.

However, there are some limitations to this study. Firstly, we have only demonstrated that MenSCs can be induced to decidualize in vitro, but it is not clear whether there were any differences between decidualization induced by MenSCs in vitro and decidualization of endometrial stromal cells in vivo. Furthermore, how to control the efficiency of the decidualization of MenSCs in vitro for clinical treatment remains a challenge. In the future, further investigation should focus on optimizing culture conditions and exploring potential molecular mechanisms to enhance the efficiency of MenSCs’ decidualization in vitro. This may provide a novel therapeutic approach for the treatment of TE patients.

## Conclusion

In conclusion, our study indicates that MenSCs can be induced into decidual cells in vitro, similar to the process in the endometrium. Both MenSCs and DSCs could increase endometrial thickness, number of glands, and fertility recovery in TE rats. This effect was mainly achieved by promoting angiogenesis and cell proliferation. After MenSC-induced decidualization, the proliferation ability of DSCs weakened, but their secretory ability was significantly enhanced. The fertility restoration capability of DSCs in TE rats was better than that of MenSCs, it may be due to its stronger ability to promote angiogenesis rather than cell proliferation. Furthermore, our study provides a new approach to restoring fertility with TE.

### Supplementary Information

ESM 1 Supplement figure Establishment of TE model with new method. (**A**) Uterine outlook and vaginal secretions of rats during estrus, there are more anucleated keratinized epithelial cells under the microscope. (**B**) Uterine outlook and vaginal secretions of rats during diestrus, there are almost no anucleated keratinized epithelial cells under the microscope. (**C**) H&E and Mason staining of normal rat uterine. (**D**) H&E and Mason staining of TE rat uterine. (**E**) Statistical analysis was performed to compare the thickness of the endometrium, gland numbers, and the ratio of fibrosis between the sham and TE groups. Values are expressed as average ± SEM of three replicates. (DOCX 369 kb)

## Data Availability

The datasets used and/or analyzed during the current study are available from the corresponding author upon reasonable request.
