# Supplementary material for: A Novel Method to Repair Thin Endometrium and Restore Fertility Based on Menstruation-Derived Stem Cell
Source: Reprod Sci. 2024 Jan 31;31(6):1662–73. doi: 10.1007/s43032-024-01458-2 (PMC11111544; doi:10.1007/s43032-024-01458-2)
Supplement: Supplementary file 1 [file 43032_2024_1458_MOESM1_ESM.docx]

Supplement

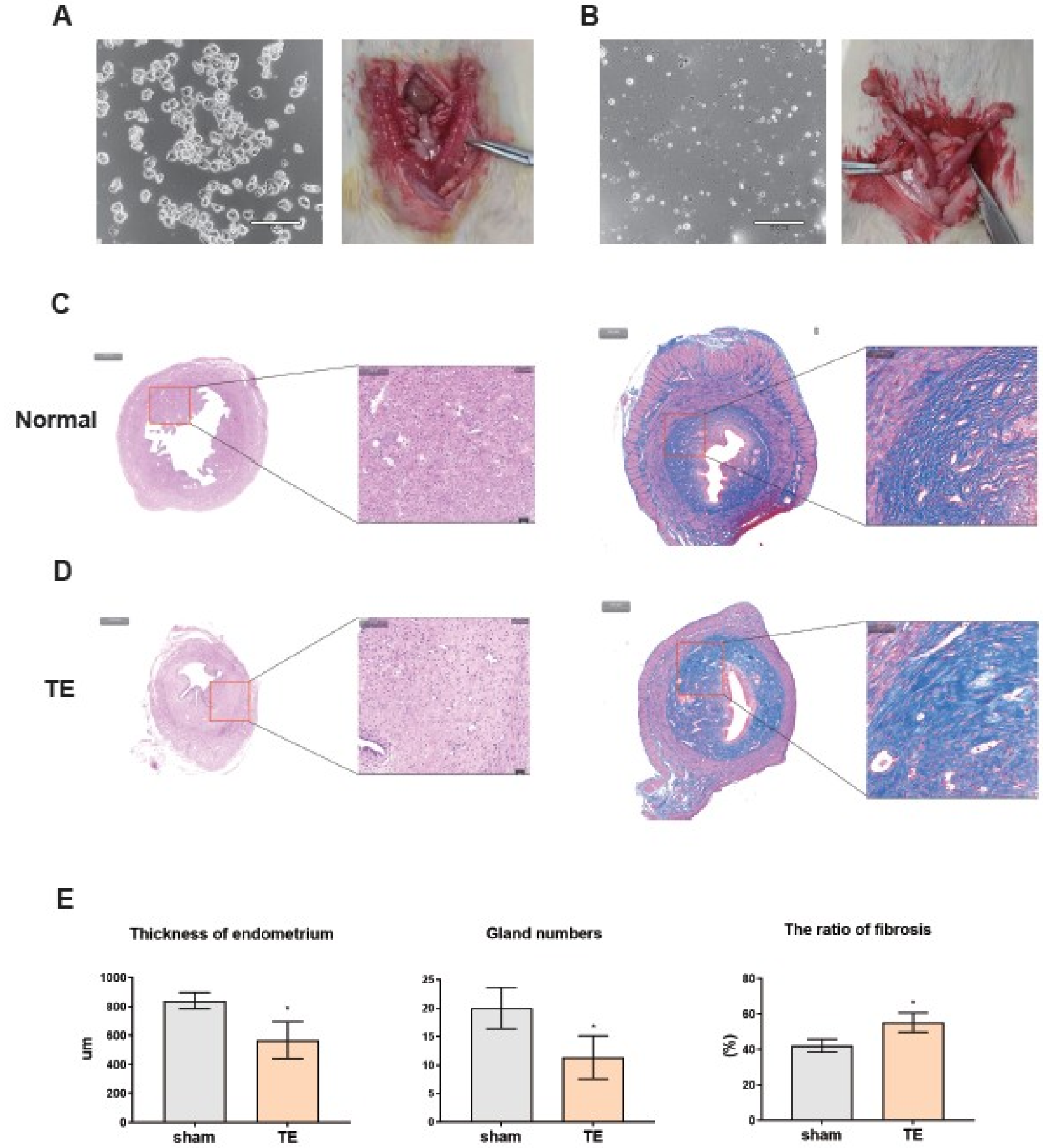
A
B
C
Normal
D
TE
E
Thickness of endometrium
um
1000
800
600
400
200
0
sham
TE
Gland numbers
25
20
15
10
5
0
sham
TE
The ratio of fibrosis
(%)
80
60
40
20
0
sham
TE
